# Biosynthesis of α-Glucosidase Inhibitors by a Newly Isolated Bacterium, *Paenibacillus* sp. TKU042 and Its Effect on Reducing Plasma Glucose in a Mouse Model

**DOI:** 10.3390/ijms18040700

**Published:** 2017-03-25

**Authors:** Van Bon Nguyen, Anh Dzung Nguyen, Yao-Haur Kuo, San-Lang Wang

**Affiliations:** 1 Department of Chemistry, Tamkang University, New Taipei City 25137, Taiwan; bondhtn@gmail.com; 2 Department of Science and Technology, Tay Nguyen University, Buon Ma Thuot 630000, Vietnam; 3 Institute of Biotechnology and Environment, Tay Nguyen University, Buon Ma Thuot 630000, Vietnam; nadzungtaynguyenuni@yahoo.com.vn; 4 Division of Chinese Materia Medica Development, National Research Institute of Chinese Medicine, Taipei 11221, Taiwan; kuoyh@nricm.edu.tw; 5 Graduate Institute of Integrated Medicine, College of Chinese Medicine, China Medical University, Taichung 40402, Taiwan

**Keywords:** α-glucosidase inhibitor, *Paenibacillus*, type 2 diabetes, acarbose, plasma glucose

## Abstract

*Paenibacillus* sp. TKU042, a bacterium isolated from Taiwanese soil, produced α-glucosidase inhibitors (aGIs) in the culture supernatant when commercial nutrient broth (NB) was used as the medium for fermentation. The supernatant of fermented NB (FNB) showed stronger inhibitory activities than acarbose, a commercial anti-diabetic drug. The IC_50_ and maximum α-glucosidase inhibitory activities (aGIA) of FNB and acarbose against α-glucosidase were 81 μg/mL, 92% and 1395 μg/mL, 63%, respectively. FNB was found to be strongly thermostable, retaining 95% of its relative activity, even after heating at 100 °C for 30 min. FNB was also stable at various pH values. Furthermore, FNB demonstrated antioxidant activity (IC_50_ = 2.23 mg/mL). In animal tests, FNB showed remarkable reductions in the plasma glucose of ICR (Institute of Cancer Research) mice at a concentration of 200 mg/kg. Combining FNB and acarbose enhanced the effect even more, with an added advantage of eliminating diarrhea. According to HPLC (High-performance liquid chromatography) fingerprinting, the *Paenibacillus* sp. TKU042 aGIs were not acarbose. All of the results suggest that *Paenibacillus* sp. TKU042 FNB could have potential use as a health food or to treat type 2 diabetes.

## 1. Introduction

Diabetes mellitus (DM) is a chronic metabolic disorder and a serious worldwide health problem. DM is commonly classified as either type 1 (insulin-dependent diabetes mellitus, IDDM) or type 2 (non-insulin-dependent diabetes mellitus, NIDDM). Among diagnosed cases of DM, more than 90% are type 2 [1]. The estimated number of people worldwide suffering from type 2 diabetes will exceed 330 million by 2030 [2]. It has been reported that type 2 diabetes can be treated with α-glucosidase inhibitors (aGIs) [3]. Even now, some commercial aGIs, such as acarbose, miglitol, and voglibose, are used for the treatment of type 2 diabetes. However, these treatments have several side effects, including diarrhea, flatulence, and abdominal discomfort, prompting the exploration of original, sufficient, and natural sources of aGIs instead.

Various natural aGIs have been extracted from medicinal plants [4]. However, these aGIs are not easy to isolate or acquire on a large scale [5,6]. Though chemically synthesized aGIs can be obtained in great amounts, they may also result in hepatic disorders and other adverse effects [7]. Biological synthesis, on the other hand, may have potential as an alternative means of acquiring natural aGIs.

So far, aGIs have been described as being produced by microorganisms, including bacteria [8,9,10,11,12,13,14,15,16] and fungi [5,17,18,19]. Among these aGIs-producing microbes, only strains of *Streptomyces* [8,9], *bacillus* [8,10,11,12,13,14], *Stenotrophomonas maltrophilia* [15], and *Actinoplanes* spp. SE-50 [16] have been studied extensively. To the best of our knowledge, there are no reports in the literature on aGIs-producing strains belonging to the genus *Paenibacillus.*

According to our recent literature review, *Paenibacillus* species have rarely been reported to produce materials detrimental to humans [20,21]. Our preceding studies revealed that *Paenibacillus* strains isolated using squid pen powder (SPP) as the sole source of carbon/nitrogen (C/N) were able to transform SPP into bioactive materials, such as exopolysaccharides by *P. mucilaginosus* TKU032 [22], and *P. macerans* TKU029 [23], antioxidants by *Paenibacillus* sp. TKU036 [20], and biosurfactants by *P. macerans* TKU029 [23]. In this study, we isolated and identified an aGI-producing strain of *Paenibacillus* sp. TKU042 which secreted acarbose-comparable aGIs in the fermented nutrient broth (FNB). The optimization of culture conditions, pH and thermal stabilities, as well as the effects of FNB on mice, were subsequently explored.

## 2. Results and Discussion

### 2.1. Isolation, Screening, and Identification of Strain TKU042

More than 600 bacterial strains were isolated from the soils of Northern Taiwan using a medium that contained 1% squid pen powder (SPP) as the sole source of carbon/nitrogen. Of these, TKU042 demonstrated the strongest inhibitory activity (97%) with an IC_50_ value of 3.9 ± 0.12 mg/mL; it was, therefore, chosen for further investigation. This potent strain was initially identified as *Paenibacillus* sp. based on 16S rDNA sequences. The name of the species was identified using an analytical profile index (API); however no match was found. Therefore, the TKU042 strain was simply labeled as *Paenibacillus* sp. Many strains of *Paenibacillus* have been reported as possessing potential industrial, agricultural, medical, and health food applications, such as the production of enzymes from *P.* *curdlanolyticus* B-6 [24], *P. barengoltzii* [25], *P. barengoltzii* [26], and *P. ehimensis* MA2012 [27], exopolysaccharides from *P. mucilaginosus* TKU032 [22] and *P. macerans* TKU029 [23], antioxidants from *Paenibacillus* sp. TKU036 [20], biosurfactants from *P. macerans* TKU029 [23], biological control agents from *P. ehimensis* MA2012 [27] and *P. polymyxa* AC-1 [28] or biofertizlizers from *P. polymyxa* [29,30]. Based on our recent literature review, the biosynthesis of aGIs by the genus *Paenibacillus* has not yet been reported.

### 2.2. Effects of the C/N (Carbon/Nitrogen) Source on aGIs Production

**Figure 1:**
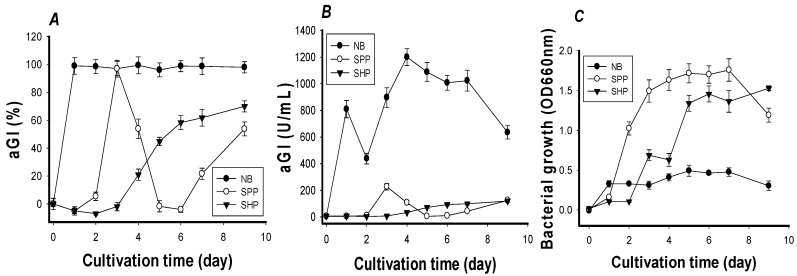
Screening C/N sources for fermentation. SPP, SHP (shrimp head powder), and NB were used as the sole sources of C/N with concentrations of 1%, 1% and 0.8%, respectively. Fermentation conditions were set at 30 °C, shaking speed of 150 rpm, 100/250 mL for the medium/flask volume ratio, and 1 mL of bacterial seed solution (OD_660nm_ = 0.38). The inhibition against yeast α-glucosidase was then tested and calculated in % (**A**) or U/mL (**B**); the growth of *Paenibacillus* sp. TKU042 was detected at OD_660nm_ (**C**). To eliminate the influence of SHP and SPP on OD_660nm_ adsorption, any residual SHP and SPP present after fermentation were removed by centrifugation at 500 rpm for 10 min. *Saccharomyces cerevisiae* α-glucosidase was used to test aGI activity.

During fermentation, the source of C/N was proposed as the significant factor in the synthesis of aGIs since it could influence the production of some aGI-related enzymes [31]. Similar phenomena were found in our previous reports, which showed SPP as the most suitable C/N source for the production of exopolysaccharides and antioxidants by isolated strains of *Paenibacillus* [20,21,22,23]. Three sources of C/N: 1% SPP, 1% shrimp head powder (SHP), and 0.8% nutrient broth (NB), were investigated for the production of aGIs by *Paenibacillus* sp. TKU042 (Figure 1). The inhibitory activities of the fermented NB, SPP, and SHP reached 100% (at day 1), 100% (at day 3), and 70% (at day 9), respectively (Figure 1A). To analyze aGI productivity, the culture supernatants were diluted appropriately to obtain aGI activity, and expressed as U/mL. As shown in Figure 1B, there were remarkable differences among the three culture supernatants. NB seemed to be the best C/N source for aGI production, showing productivity (1200 U/mL at day 4) approximately 5.3- and 10-fold higher than those of SPP (220 U/mL at day 3) and SHP (120 U/mL at day 6), respectively. Throughout fermentation, the cell growth of *Paenibacillus* sp. TKU 042 was also monitored by measuring the absorbance of the cell solution at 660 nm. As shown in Figure 1C, there was no relationship between cell growth and aGI productivity.

Soybean has been widely used in many studies as the sole source of C/N to synthesize aGIs [8,11,12,17,18]. To enhance aGI production, the soybean-containing medium has been supplemented with various carbohydrates. For example, *Streptomyces lavendulae* GC-148 produced an aGI in medium containing 2% glucose and 1.5% soybean [8], and the *Bacillus* species required 4% soybean peptone supplemented with 5% carbon source (glucose, galactose, lactose, or sorbitol) [11]. In this study, *Paenibacillus* sp. TKU 042 used NB for aGI production, diverging from the other reports.

### 2.3. Optimization of Culture Conditions

**Figure 2:**
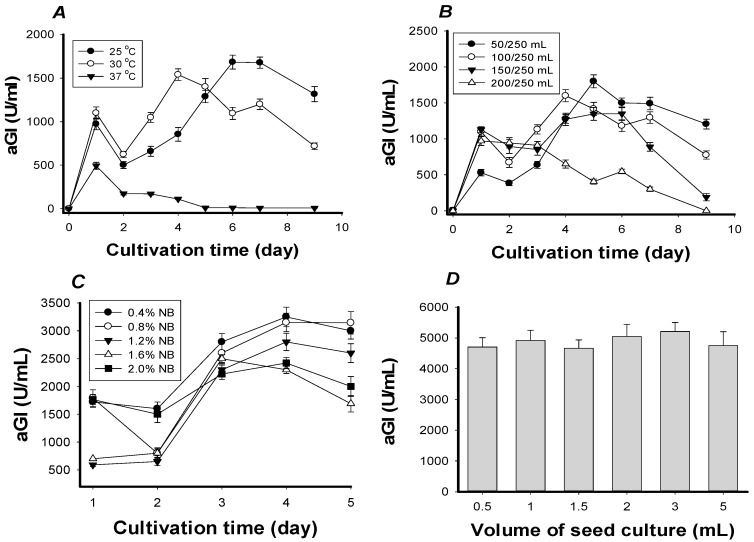
The effects of some parameters on aGIs production. (**A**) The effect of incubation temperature on aGI activity: Fermentation was conducted using 150 rpm shaking speed, a medium/flask volume ratio of 100/250 mL, 0.8% NB (C/N source), and 1 mL of seed culture (OD_660nm_ = 0.38); (**B**) the effect of medium/flask volume ratio on aGI productivity: fermentation was conducted at 30 °C, with a shaking speed of 150 rpm, and using 0.8% NB (C/N source), and 1 mL of seed culture (OD_660nm_ = 0.38); (**C**) the effect of NB concentration on aGI productivity: fermentation conducted at 30 °C, with a shaking speed of 150 rpm, a medium/flask volume ratio of 100/250 mL, and 1 mL of seed culture (OD_660nm_ = 0.38); and (**D**) the effect of seed culture volume on aGI productivity: fermentation was conducted at 30 °C, with a shaking speed of 150 rpm, a medium/flask volume ratio of 100/250 mL, and using 0.8% NB (C/N source). *Saccharomyces cerevisiae* α-glucosidase was then used to analyze the aGIA.

NB was confirmed as the best source of C/N, and chosen to establish optimal parameters, including cultivation temperature, culture volume, concentration of NB, and the amount of seed culture inoculated (Figure 2).

The results showed that aGI productivity was highest on the sixth day of cultivation at 25 °C (1700 U/mL) and the fourth day of cultivation at 30 °C (1550 U/mL) (Figure 2A). Taking cultivation time into account, 30 °C was selected to study the effects of culture volume. As shown in Figure 2B, aGI productivity was highest on the sixth day of cultivation in 50 mL-medium (1800 U/mL) followed by the fourth day of cultivation in 100 mL-medium (1600 U/mL). After considering the recovered volume of culture supernatant, 100 mL-medium (at 30 °C) was selected to investigate the effects of NB concentration on aGI productivity. As seen in Figure 2C, the highest aGI productivity was found on the fourth day when either 0.4% or 0.8% NB was used. 0.8% NB was ultimately selected for further research since aGI productivity declined slightly after day 4 when 0.4% NB was used. The inoculated volume of the seed culture had no significant effect on aGI production (Figure 2D).

Overall, aGIs were effectively produced by *Paenibacillus* sp. TKU042 in the 0.8% NB-containing medium (100 mL of medium in a 250 mL-Erlenmeyer flask) at 30 °C and an initial pH of 6.85, using a reciprocal shaker at 150 rpm for 4 days. The culture conditions before and after the optimization study are summarized in Table 1; in short, the IC_50_ and productivity of *Paenibacillus* sp. TKU 042 aGIs were increased approximately 23-fold after optimization.

### 2.4. Specific aGI Activity and Antioxidant Activity of FNB

To evaluate the potential of *Paenibacillus* sp. TKU 042 aGIs as anti-diabetic drugs, the inhibitory specificity of FNB was tested against eight kinds of commercial enzymes. As shown in Table 2, FNB showed strong inhibitory activity against bacterial (*Bacillus stearothermophilus*) α-glucosidase (IC_50_ = 36.7 ± 1.7 µg/mL), yeast (*Saccharomyces cerevisiae*) α-glucosidase (IC_50_ = 81 ± 4.3 µg/mL), and rat α-glucosidase (IC_50_ = 101 ± 5.1 µg/mL), but had a weaker response against rice α-glucosidase (IC_50_ = 508 ± 17 µg/mL). On the other hand, the anti-diabetic drug acarbose demonstrated better inhibitory activity against α-glucosidases from bacteria (0.012 ± 0.001 µg/mL), rice (2.92 ± 0.67 µg/mL), and rat (107 ± 2.5 µg/mL), but showed weaker inhibitory activity against yeast α-glucosidase (1395 ± 5 µg/mL).

α-Glucosidase from *S. cerevisiae* has been commonly used to evaluate aGI activity in many studies [18,32,33,34]. Compared to yeast and other sources of α-glucosidases, rats may be a more valuable resource since the animal enzyme is closer to that of humans [32]. In this study, both FNB and acarbose inhibited α-glucosidases originating from rats and yeast. Among them, FNB showed much stronger aGI activity than acarbose against α-glucosidase from yeast but approximately the same amount of inhibition against rat α-glucosidase. Therefore, FNB could be a potential candidate for α-glucosidase inhibition.

α-Amylase inhibitors have been reported to be a useful treatment for type 2 diabetes [35]. Consequently, the inhibitory activities of FNB against α-amylases were also studied. As shown in Table 2, FNB showed no inhibitory activity against porcine pancreatic and *B. subtilis* α-amylases. Similarly, FNB demonstrated no inhibitory activity against proteases from bromelain or papain. It is well-known that there are several proteases in the gastrointestinal tract to help ingest proteins. Since FNB showed no inhibitory activity against these tested proteases, it may have the potential to avoid disturbing the ingestion of proteins by the use of aGIs.

Several previous studies [20,22] suggested that antioxidant compounds could be produced by the genus *Paenibacillus.* Therefore, the antioxidant property of FNB was also tested, using the 2,2-diphenyl-1-picrylhydrazyl (DPPH) radical scavenging activity assay mentioned in the experimental methods section, to provide more bioactive proof of FNB’s usefulness. The antioxidant activity was calculated as both a percentage and IC_50_ value. α-Tocopherol, a commercial antioxidant compound, showed stronger activity than FNB, generating IC_50_ values of 0.0247 ± 0.0012 and 2.23 ± 0.14 mg/mL, respectively. However, FNB can achieve the same amount of activity (approximately 100%) as α-tocopherol. Overall, FNB could be used as a health food due to its valuable bio-functions, including its potential for α-glucosidase inhibition and acceptable antioxidant activity.

### 2.5. Confirmation that aGIs Contained in FNB Were Produced during NB Fermentation

**Figure 3:**
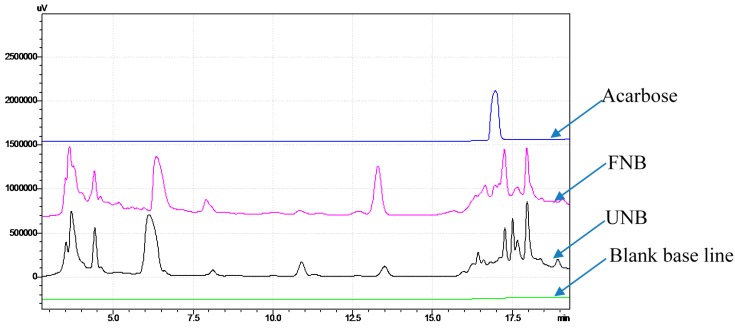
The HPLC finger prints of unfermented and fermented NB. Analysis conditions: mobile phase: 0–5′ (1% Acetonitrile (ACN), 5–20′ (1%–10% ACN), 20–25′ (10%–40% ACN), 25–40′ (40%–50% ACN); Ultraviolet (UV) detector: 240 nm; flow rate 0.8 mL/min, column temperature: 25 °C, using C18 column.

The same concentrations (20 mg/mL) of unfermented NB (UNB) and fermented NB (FNB) solutions were analyzed by HPLC. The differences in the HPLC finger prints of NB before and after fermentation are clearly observed in Figure 3. After fermentation, some major peaks disappeared (the peaks at retention times of 6, 13.5, and 17.5 min) or reduced their area (the peak at 11 min), while some new peaks appeared at 6.5 and 13.2 min, and the peak at 17.3 min, enhanced its area. UNB was tested for α-glucosidase inhibition but no activity was observed. The differences in both the HPLC finger prints and the inhibitory activity of UNB and FNB suggest that the aGIs were produced by fermentation, since none existed in the NB beforehand. Each fraction of aGI activity in FNB was analyzed on the HPLC finger print; three fractions showed aGI activity (≥85% at the concentration of 200 µg/mL). The isolation and identification of these aGIs will eventually be conducted. The HPLC finger print of acarbose was also analyzed; based on the difference in retention time, we confirmed that the active compound in FNB aGIs was not acarbose.

### 2.6. The Thermal and pH Stabilities of FNB aGIs

**Figure 4:**
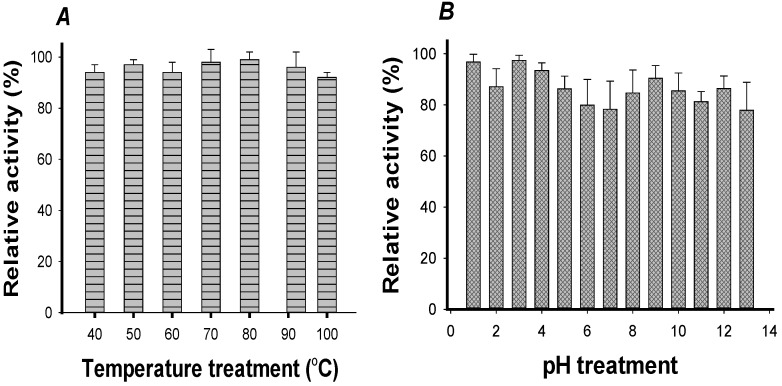
Thermal and pH stability of FNB. Thermal stability (**A**) was tested by treating FNB for 30 min at 40–100 °C and pH 6.8; the residual aGI activity was analyzed at 37 °C; The pH stability (**B**) was tested by treating FNB to a pH range of 1–13 at 37 °C for 30 min then evaluating the residual aGI activity at pH 6.8.

The thermal stability of FNB aGIs was determined by first treating FNB at 40–100 °C for 30 min, and then testing the residual aGI activity. FNB aGIs were strongly thermostable at all temperatures, with relative activities greater than 95% (Figure 4A). They maintained a relative activity of 92% even when heated at 100 °C for 30 min. The high thermal stability of FNB aGIs suggests they may have the potential to remain active for a long time.

pH stability is also an important factor. After treating FNB with a large range of pH values at 37 °C for 30 min, the residual aGI activity was analyzed at pH 6.8. As shown in Figure 4B, aGI activity remained at 87%–96% after 30 min pre-incubation at pH ranges from 1–13. It is well-known that the stomach is very acidic; therefore, potential aGIs should be stable and able to work well in the acidic environments of the gastrointestinal tract. Bacteria-produced aGIs that show thermal and pH stability are rarely reported. In the previous reports [32,35], aGIs from ELC, a methanolic extract from a medicinal plant (*Euonymus laxiflorus* Champ), were reported to be heat-stable at 97 ± 3 °C (heating 30 min) with a relative activity of 90%, demonstrating the same thermal stability as FNB. However, the relative activity of this extract (50%) was lower than that of FNB (80%–93%) in the acidic environment (pH 4–6).

### 2.7. The Effects of FNB on Reducing Plasma Glucose in the Mouse Model

**Figure 5:**
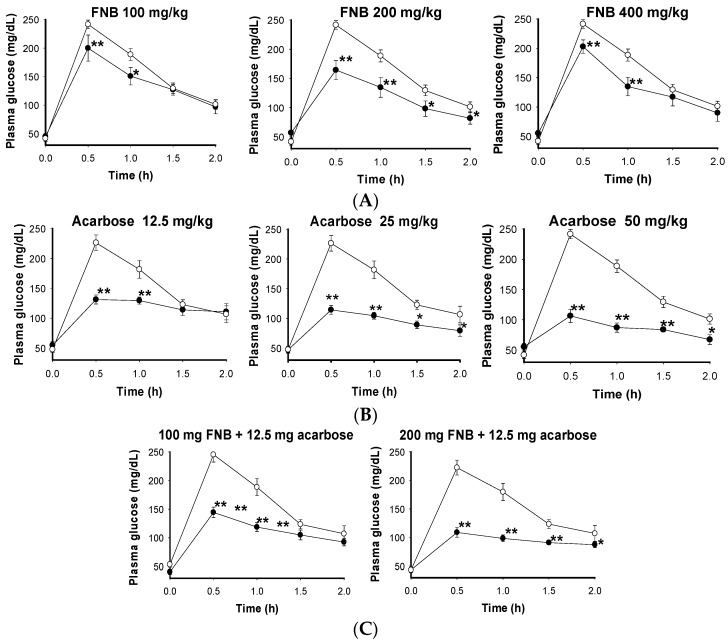
Effects of FNB and acarbose, alone or in combination, on the increase in plasma glucose levels following oral sucrose loading in ICR mice. (**A**) Oral FNB administration; (**B**) Oral acarbose administration; (**C**) Combined FNB and acarbose administration. FNB (100, 200, and 400 mg/kg bw), acarbose (12.5, 25 and 50 mg/kg bw), and a combination of FNB (100 and 200 mg/kg bw) and acarbose (12.5 mg/kg bw) were administered to mice (●, *n* = 8). In the control groups (◦, *n* = 8), distilled water was administered. After loading the sample (or acarbose or distilled water) for 25 min, sucrose solution was orally administered at a concentration of 3 g/kg bw; blood was sampled and measured 0.5, 1, 1.5 and 2 h, thereafter. Statistical analyses were conducted using the Least Significant Difference (LSD) test. The significant differences for each experimental mean (in the comparison with its control) are illustrated with (*) at *p* < 0.05 and (**) at *p* < 0.01.

Three doses of FNB (100, 200, and 400 mg/kg) were used on ICR mice to evaluate their impact on plasma glucose. As shown in Figure 5A, significant reductions in plasma glucose were observed 0.5 and 1 h after sucrose loading for 100 and 400 mg/kg.

The FNB with 200 mg/kg body weight (bw) was ultimately the best, as it reduced blood sugar for 1.5 h. This result is comparable with a dose of acarbose at 25 mg/kg, but better than acarbose at 12.5 mg/kg (Figure 5B).

In the combined FNB and acarbose group, two doses of FNB (100 and 200 mg/kg) and one dose of acarbose (12.5 mg/kg) were used (Figure 5C). The combination of FNB at 100 mg and acarbose at 12.5 mg had no effect compared to acarbose (12.5 mg/kg) administration alone. On the other hand, the combination of FNB at 200 mg/kg and acarbose at 12.5 mg/kg was much better at reducing blood glucose than acarbose alone at 12.5 mg/kg, slightly higher than acarbose at 25 mg/kg, and comparable to arcabose at 50 mg/kg. The number of mice with diarrhea was also recorded. No ill effects were observed in the control, FNB at 100, 200, or 400 mg, acarbose at 12.5 mg or the combination of FNB (100, 200 mg) with acarbose at 12.5 mg. However, for the groups who received 25 and 50 mg of acarbose, diarrhea was observed in 3/8 mice (37.5%) and 5/8 mice (62.5%), respectively.

These results show that oral FNB loading can reduce plasma glucose levels in mice, and the combination of FNB (200 mg/kg) and acarbose (12.5 mg/kg) can produce approximately the same effect as acarbose alone at 25 and 50 mg. As such, the acarbose dosage can be reduced two- to four-fold, resulting in reduced side effects.

## 3. Materials and Methods

### 3.1. Materials

Nutrient broth was purchased from Creative Life Science Co., Taipei, Taiwan, squid pens were acquired from Shin-Ma Frozen Food Co. (Yilan, Taiwan), and shrimp head power (SHP) was obtained from Fwu-Sow Industry (Taichun, Taiwan). Rat α-glucosidase (intestinal acetone powders from rat) was purchased from Sigma Aldrich, Singapore. Acarbose, *Saccharomyces cerevisiae* α-glucosidase, *Bacillus stearothermophilus* α-glucosidase, and 2, 2-diphenyl-1-picrylhydrazyl (DPPH) were purchased from Sigma Chemical Co., St. Louis, MO, USA. Rice α-glucosidase (Type 4) and porcine pancreatic α-amylase (Type VI-B) were purchased from Sigma Aldrich, St. Louis, MO, USA. The proteases of bromelain and papain were obtained from Challenge Bioproducts Co., Ltd., Yunlin, Taiwan. When possible, reagents, solvents and other common chemicals were obtained at the highest grade.

### 3.2. Measurement of Rat α-Glucosidase Inhibition

To determine rat intestinal α-glucosidase inhibition, the techniques from the previous report were followed [36], with modifications. One hundred mg of rat-intestinal acetone powder was suspended in 4 mL of 0.1 mol/L potassium phosphate buffer (NPB), pH 7. The suspension was sonicated several times for 12 s at 4 °C and then centrifuged for 20 min at 10,000× *g*. The residues were twice suspended with 3 mL of 0.1 mol/L NPB at pH 7, as described above. The resulting supernatant was mixed together and dialyzed for 12 h at 4–6 °C, and then used for the assay. The α-glucosidase, sample solutions and buffer were pre-mixed at volumes of 50, 50 and 100 μL, respectively. After pre-incubation at 37 °C for 20 min, the reaction started when 50 μL of *p*-nitrophenyl glucopyranoside (10 mmol/L) was added to the mixture. After 20 min at 37 °C, 100 μL Na_2_CO_3_ solution (1 mol/L) was added to terminate the reaction. The absorbance of the final mixture was measured at 410 nm [37] and used to calculate inhibition (%) using the following equation:

α-glucosidase inhibitory activity (%) = (A − B)/A × 100

where “*A*” is the absorbance of the control (buffer was used instead of sample with the same volume) and “*B*” is the absorbance of the final mixture containing the sample and α-glucosidase [38]. The IC_50_ value was defined and determined as per the previous study [32]. The α-glucosidase and the samples were prepared in 0.1 mol/L potassium phosphate buffer (pH 7).

General α-glucosidase and α-amylase inhibition assays were carried out as described in our previous papers ([32,35], respectively). Acarbose dissolved in the same buffer (pH 7) was also analyzed as a control.

### 3.3. DPPH Radical Scavenging Activity Assay

One hundred and twenty microliters of each sample (at different concentrations) was mixed with 30 µL of a methanolic solution containing 0.75 mM DPPH radicals in the well of a plate with 96 wells. The mixture was kept in the dark for 30 min before absorbance at 517 nm was measured [39]. Radical scavenging activity was calculated using the following formula:

Antioxidant activity (%) = [(AB − AA)/AB] × 100

where *AB* and *AA* stand for absorption of the blank sample (*t* = 0 min) and absorption of the tested sample solution (*t* = 30 min), respectively. α-Tocopherol dissolved in MeOH was also analyzed as a control.

### 3.4. Isolation and Screening of aGI-Producing Strains

About 60 soil samples were randomly collected from Northern Taiwan. For convenience, half of the soil samples were collected from the campus of Tamkang University (Tamsui, New Taipei City, Taiwan). Ten milliliters of sterile distilled water was added to 1 g of ground soil and stirred. Half a milliliter of the soil suspension was then added to medium containing 0.1% K_2_HPO_4_, 0.05% MgSO_4_·7H_2_O, and 1% squid pen powder (SPP), supplemented with 1.5% agar in a plastic Petri dish containing 10 mL of medium (pH 7.2). After 1–3 days of incubation at 37 °C, the single bacterial colonies that appeared were sub-cultured several times on the same medium.

All of the isolated strains were incubated in a 250-mL Erlenmeyer flask with 50 mL of the same medium, but without agar. Fermentation was performed at 37 °C and a 150 rpm shaking speed. After 1–4 days of fermentation, the culture was centrifuged at 500 rpm for 10 min to remove the medium residue. The solution was then centrifuged again at 4000 rpm for 20 min to separate the cells from the culture supernatant. The supernatant was then used to test yeast α-glucosidase inhibitory activity. The cells were dissolved in the same volume of distilled water as the supernatant and measured at 660 nm to detect bacterial growth. The screening of active bacteria was based on the inhibitory activity of the supernatant.

### 3.5. Optimization of Culture Conditions for Synthesis of aGIs

Three sources of C/N (1% SPP, 1% SHP, and 0.8% NB) were used to cultivate *Paenibacillus* sp. TKU042; conditions were set at 30 °C, a shaking speed of 150 rpm and a 100/250 mL medium/flask volume ratio for nine days. NB appeared to be most suitable for synthesizing aGIs and was chosen to test optimization of specific parameters, including concentration of NB (0.4%, 0.8%, 1.2%, 1.6%, 2.0% *w*/*v*), medium volume (50, 100, 150, and 200 mL), culture temperature (25, 30, and 37 °C) and the amount of seed culture (0.5, 1, 1.5, 2, 3, and 5 mL of bacterial seed solution at OD_660nm_ = 0.38). The culture supernatant obtained was centrifuged again at 4000 rpm for 20 min, then used to test yeast α-glucosidase inhibitory activity.

### 3.6. Measurement of Inhibitor Stability

The pH stability of the sample was determined as per the methods described in our previous paper [35]. To determine thermal stability, the sample was exposed to a temperature range of 40–100 °C for 30 min before testing for inhibition against yeast α-glucosidase, using the general α-glucosidase inhibition assay [32].

### 3.7. Experimental Animal Protocol

Seventy-two seven week-old male ICR mice obtained from The National Laboratory Animal Center (No. 128, Sec. 2, Academia Rd., Nangang Dist., Taipei City 11529, Taiwan) were randomly divided into nine groups (8 mice per group). All had equivalent mean plasma glucose levels and body weights, including the control group (orally administered with distilled water only). Three experimental groups received oral FNB (100, 200, or 400/kg), three experimental groups received oral acarbose (12.5, 25 or 50 mg/kg), and two experimental groups received both FNB and acarbose (100 mg of FNB combined with 12.5 mg of acarbose, or 200 mg of FNB combined with 12.5 mg of acarbose). This animal study was conducted in accordance with the guidelines and approval of the Institutional Animal Care and Use Committee of the National Research Institute of Chinese Medicine, Ministry of Health and Welfare. (IACUC No.104-706-1, 29 December 2014).

The sucrose tolerance test was performed following the methods described in the previous report [40], with slight modifications. Mice were fasted for 16 h and were then given different concentrations of distilled water (control group), acarbose, or mixtures of the solutions described in detail above for 20 min. A sucrose solution was orally administered at 3 g/kg bw and blood was sampled and measured after 0.5, 1, 1.5 and 2 h.

Statistical Analysis Software (SAS-9.4, provided by SAS Institute Taiwan Ltd, Minsheng East Road, section 2, Taipei, Taiwan 149-8) was used to analyze the significant differences of the calculated IC_50_ values, the α-glucosidase inhibition (%) and the plasma glucose levels.

## 4. Conclusions

Of more than 600 bacterial strains isolated from Taiwanese soils, *Paenibacillus* sp. TKU042 showed the best potential as a source of aGIs. This bacterium produced aGIs and antioxidants when NB was used as the sole source of C/N. The aGIs of the fermented product (FNB) showed higher inhibitory activity than acarbose against rat and yeast α-glucosidases. The FNB aGIs also showed high thermal and pH stability, even when pre-incubated at 100 °C or exposed to acidic conditions (pH 1) for 30 min. FNB also had an acceptable effect on reducing plasma glucose in mice. All of these results suggest that NB is a potential carbon/nitrogen source for producing aGIs using *Paenibacillus* sp. TKU 042. Furthermore, the aGIs produced might be useful candidates for treating type 2 diabetes and obesity, as well as future use as a health food.

## Figures and Tables

**Table 1:** Comparison of culture conditions before and after optimization.

Compared Factors	Before Optimization	After Optimization
C/N source	SPP	NB
Cultivation temperature (°C)	37	30
C/N Concentration (%)	1	0.8–1.2
Medium/flask volume ratio	2/5	2/5
Seed culture (%)	1	1
Inhibition (IC_50_ μg/mL)	3900 ± 120	81 ± 4.3
aGIs Productivity (U/mL)	220	5000

**Table 2:** Specific inhibitory activity of FNB and acarbose against enzymes.

Enzyme	*n*	Inhibition of FNB		Inhibition of Acarbose	
		IC_50_ (µg/mL)	Maximum Inhibitory Activity (%)	IC_50_ (µg/mL)	Maximum Inhibitory Activity (%)
*S. cerevisiae* α-glucosidase	3	81 ± 4.3 ^c,d^	92 ± 3.2	1395 ± 5 ^a^	63 ± 2.5
Rat α-glucosidase	3	101 ± 5.1 ^c^	95 ± 2.1	107 ± 2.5 ^c^	89 ± 2.4
*B. stearothermophilus* α-glucosidase	3	36.7 ± 1.7 ^c,d^	98.2 ± 1.3	0.012 ± 0.001 ^d^	100 ± 1.2
Rice α-glucosidase	3	508 ± 17 ^b^	94 ± 2.3	2.92 ± 0.67 ^d^	100 ± 1.4
Porcine pancreatic α-amylase	3	-	-	ND	ND
*B. subtilis* α-amylase	3	-	-	ND	ND
Papain protease	3	-	-	ND	ND
Bromelain protease	3	-	-	ND	ND

(-) no inhibition; ND: not determined; CV = 12.74; LSD_0.01_ = 86.385; *n* = 3: triplicates of each experiment. Mean of IC_50_ values with the different letters are significantly different based on *t* test ranking.
